# The Impact of Host Factors on Management of Hepatitis C Virus

**DOI:** 10.5812/hepatmon.709

**Published:** 2012-04-30

**Authors:** Mahmoud Aboelneen Khattab, Mohammed Eslam

**Affiliations:** 1 Department of Internal Medicine, Minia University, Minia, Egypt

**Keywords:** Host Factors, Liver Diseases, Hepatitis C Virus

## 1. Introduction

Hepatitis C virus (HCV) was identified in 1989 as an enveloped virus with a 9.6-kb single-stranded RNA genome and a member of the Flaviridae family, genus Hepacivirus. It is characterized by a high spontaneous mutation rate with an estimated frequency of 1.4–1.9 × 10(³) mutations per nucleotide per year [1][2]. As a result, HCV exists as a heterogeneous group of viruses, sharing approximately 70% homology. On the basis of nucleotide sequence homology, HCV has been classified into no fewer than 6 major genotypes and a series of subtypes [3]. HCV is among the leading causes of chronic liver disease worldwide and affects approximately 170 million people [4]. The severity of the disease that is associated with HCV infection varies from asymptomatic, chronic infection to cirrhosis and hepatocellular carcinoma [5][6]. Combination therapy with pegylated interferon alpha and ribavirin (Peg-IFN-α/RBV) for 24–48 weeks is the current standard of care in CHC patients [7]. Unfortunately, more than 40% of patients with chronic HCV infection will fail to control viral replication with therapy (nonresponders) or will experience a relapse when therapy is stopped (relapsers); in addition, it is physically and economically demanding. Thus, it is crucial to understand the mechanisms of nonresponse to overcome it and identify factors that can help predict the chances of a patient responding to the treatment. The molecular mechanisms that underlie the failure of standard therapy are not well understood, but evidence indicates that both viral and host factors are involved. The focus of interest of this review is to present the host factors that are associated with response to the current treatment against HCV. Several host factors are implicated in modulating the effectiveness of interferon therapy for the treatment of HCV infection [8]. Likely, these host factors play an equally important role in modulating the efficacy of interferon therapy. Therefore, an understanding of how these factors influence interferon therapy may identify therapeutic targets to improve the efficacy of interferon therapy [9]. Host factors that are responsible for responsiveness to HCV therapy with Peg-IFN-α/RBV include host genetic factors, age, ethnicity, gender, cirrhosis, weight (BMI), steatosis, insulin resistance (IR), and type 2 diabetes mellitus (T2DM).

## 2. Host Genetic Factors

The mechanism of action of IFN has been characterized, and the key points that have been identified are: a) its interaction with IFN-α receptor; b) Janus kinase (JAK) signal transducer activity; c) STAT phosphorylation; d) synthesis of antiviral proteins, such as 2’-5’-oligoadenylate synthetase enzyme (5`2` OAS), myxovirus resistance A (MxA) protein induced by IFN works like guanosine-5’-triphosphate (GTPase) to achieve its antiviral effect. The identification of new molecular markers that are related to the response to Peg-IFN/RBV represents an important rising concern. Our understanding of the genetic basis for this response is evolving. Several SNPs from candidate genes have been identified to associate with achievement of SVR in patients who receive Peg-IFN/RBV treatment. Regulatory genes of IFN antiviral activity, immune response genes, and genes that are implicated in obesity or IR have been analyzed. Intracellular MxA seems to be the most specific marker of the antiviral activity of IFN [10]. The -88T SNP in the MxA gene was found to be associated with lower MxA activity. In patients with low viral load, sustained viral response (SVR) was significantly higher (62%) in -88T patients than in patients bearing the -88A allele (32%) [11]. The 5`2` OAS plays a major role in the clearance of the virus. However, some studies that included an analysis of the GG genotype (in the 3’UTR region) did not predict SVR [12]. Lastly, a tandem repeat of 3 nucleotides in the protein kinase R gene, considered “large” when containing > 9 repeats, has been found to be associated with SVR. The large/large polymorphism was seen more often in patients achieving SVR than in nonresponders (89.4% vs. 71.8% ; P = 0.017) [13]. Apolipoprotein E has been implicated in the entry of HCV into the cell via the LDL receptor. In a cohort of 506 patients treated with Peg-IFN/RBV, the €4 allele was found to be associated with poorer response in patients with genotype 1 (30% vs. 42% ; P < 0.05) [13]. Several polymorphisms in proinflammatory cytokines have been examined as candidate genes in the prediction of SVR. The biallelic polymorphism in tumor necrosis factor (TNF) (-238 and -308) does not appear to be associated with SVR [14]. TGF-1 and interleukin-10 polymorphisms have been strongly related to achieving an SVR. The -29C/C genotype in TGF-beta1 promotes resistance to Peg-IFN/RBV treatment [15]. In a multivariate analysis of 105 patients who were treated with Peg-IFN/RBV, HLA class I B44 was seen to be independently associated with an improved SVR to combined Peg-IFN/RBV, together with the viral non-1 genotype.

Although the potential value of these well-selected candidate genes and relevance of some of their associations were confirmed after a multivariate analysis, the majority of them had a minor impact on clinical practice, and they have not been included in the daily management of patients with chronic hepatitis C. Several pharmacogenetic/genomewide association studies (GWASs) on response to HCV treatment have identified a relationship between several polymorphisms in interferon lambda (IFN-X), transduced from the IL28B gene (19q13) region, and SVR. Ge et al. [19] analyzed 1137 patients with HCV genotype 1 infection and identified several SNPs near the IL-28B gene on chromosome 19 that were significantly more common in responders than nonresponders. Moreover, the distributions of this CC genotype in were strongly related to SVR in Asians, Europeans, Hispanic, and African-Americans. These results have since been confirmed in many series [17][18]. A study by Thomas et al. [19] reported that the same IL-28B variant that was described by Ge and coworkers was associated with spontaneous clearance of HCV. A strong association of rs12979860 with both EVR and SVR in IFN-naive patients who were treated with Peg-IFNα- 2a/RBV has also been reported [20]. Further, this polymorphism has been strongly associated with the possibility of achieving SVR in patients who have been infected by genotype 1, without rapid virological response [21]. A recent meta-analysis confirmed the association between genotype CC rs12987960 and SVR (OR:4.5, 95% CI: 2.8-7.3) [22]. Conversely, the role of the IL28B polymorphism is unknown in patients with a non-1 chronic hepatitis C genotype regarding its association with SVR rates. The IL28B polymorphism rs8099917 G is not associated with higher SVR rates. In 230 patients with genotype 2 or 3 who received Peg-IFN/RBV, the CC genotype predicted SVR in patients without rapid virological response (clearance of the virus after 4 weeks of treatment) but not in the overall cohort [23]. In a multivariate analysis of HCV-4 patients, baseline viral load, fibrosis, and the IL28 T allele (OR: 0.124, 95% CI.: 0.030-0.505) were significantly associated with SVR [24]. However, the strongest predictor of final outcome was RVR (OR: 26.00; 95% CI: 7.148-94.545, P < 0.0001). If RVR was included in the multivariate model, only the RVR and fibrosis score remained significant. Thus, determining the IL28 polymorphism may not be useful in selecting patients with HCV-4 for abbreviated treatment schedules [24]. However, larger prospective studies are warranted before we can determine the predictive value of IL28B in HCV-4.

IL28 polymorphisms are highly promising genetic markers, but the role of genetic alterations in patients who are infected by non-1 genotypes should be further demonstrated. On the other hand, from a clinical point of view, approximately one-third of patients bearing the CC genotype (rs12979860) did not achieve SVR, while nearly half of CT heterozygotes and one-third of TT homozygous individuals could achieve an SVR when treated with Peg-IFN/RBV [22]. Thus, genetic factors that modulate (positively or negatively) the effect of this polymorphism on sustained responses warrant further exploration. Other genetic markers could explain this gap between IL28B genotype and sustained response [25].

## 3. Age

Patient age is another factor that is associated with responsiveness to Peg-IFN-α/RBV therapy in chronic HCV infection. Generally, it is believed that younger individuals (usually < 40 years of age) respond better to IFN-α treatment than older persons [26][27]. In a recent study evaluating the predictors of SVR for HCV genotype 1 patients who were enrolled in 2 phase III studies with PEG-IFNα-2a, Reddy and coworkers [26] showed that patients aged > 50 years had poorer responses to combined therapy than patients aged < 50 years. Interestingly, the lower SVR rates in older patients resulted primarily from a higher relapse rate. The importance of considering age in a patient who is initiating therapy for HCV has been highlighted by a recent study by Davis and coworkers [28], who developed a multicohort natural history model for predicting disease outcomes and the benefits of therapy in HCV-infected patients. The obvious explanation is that older HCV patients are likely to have more advanced liver disease, such as fibrosis and cirrhosis (themselves predictors of poor virological responses). Impairments in cellular, humoral, and innate immunity in the elderly may be another important factor that is responsible for decreasing successful responses to IFN-α treatment in older patients [29].

## 4. Metabolic Syndrome

Chronic hepatitis C (CHC) can be considered not only a viral disease but also a special type of metabolic disease. CHC interacts with lipid metabolism, leading to steatosis; impairs glucose metabolism, leading to IR and T2DM; and is associated with an increased risk of carotid atherosclerosis [30]. This combination of favorable lipids and diabetes is unusual, as the conventional metabolic syndrome, a constellation of risk factors for atherosclerosis, includes, among others, an atherogenic lipid profile, glucose intolerance, and IR [31].

## 5. Insulin Resistance

**Figure 1:**
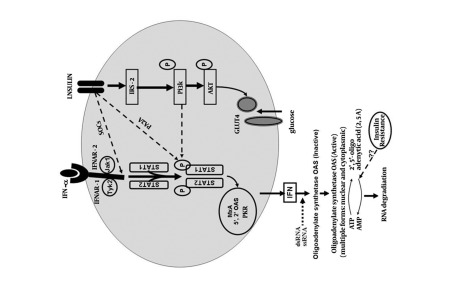
Interaction Between Insulin and Interferon-α Signaling Pathway.(SOCs, suppressor of cytokine signaling; PA2A, protein phosphatase 2A; PI3K, phosphatidil-inositol 3-kinase; JAK, janus kinase; STAT, signal transduction and activator of transcription; TYK2, tyrosine kinase 2; Dotted lines, represent inhibition; continuous lines, represent activation. Adapted from Khattab MA with permission [9].)

There is a complex interaction between HCV infection and insulin sensitivity [32]. Hepatitis C core protein promotes the degradation of insulin receptor substrate-1 and induces IR. IR, obesity, and steatosis are also associated with a higher risk of fibrosis progression [33][34]. Increasing levels of IR are associated with reduced rates of rapid virological response [35][36] and SVR in patients with HCV genotypes 1, 2, 3, and 4 infections who are treated with a combination of Peg-IFN-α and RBV [33][37][38][39]. However, the mechanisms of IR-induced IFN resistance are not completely understood. Intracellular factors that are dysregulated by HCV and are responsible for the insulin-resistant phenotype may have additional effects, as they are also involved in regulating IFN-α signaling (Figure 1). These factors include some members of the suppressor of cytokine signaling (SOCS) family [40][41][42] and protein phosphatase 2A (PP2A) [43]. Recent studies have indicated that SOCS-3 plays a crucial role. SOCS-3 is known to suppress insulin signaling through the ubiquination of insulin receptor substrates 1 and 2 (IRS) and cause IR [38]. Recently, Vanni et al. reported an increase in intrahepatic SOCS-3 mRNA expression with hepatic IR [44]. SOCS-3 also inhibits the IFN-induced JAK signal transducer and suppresses the expression of antiviral proteins, including 2,5-OAS and MxA [45][46]. Hepatic expression of SOCS-3 increases significantly in nonresponder patients compared with responders [46]. Specifically, a genetic polymorphism of SOCS-3 (the 4874 AA genotype) is strongly associated with nonresponse to antiviral therapy [40]. Further, the reduced hepatic response to IFN in HCV-infected chimpanzees is mediated via the activation of SOCS-3 [47][48], and hepatic expression of SOCS-3 predicts the outcome of antiviral therapy [46]. IR is known to increase hepatic lipid synthesis [49]. Since the lipid droplet is an important organelle for HCV replication [50], the accumulation of hepatic lipid droplets may increase HCV replication and result in poor responses to antiviral treatment. As such, host and viral factors are involved in the development of IR and, as a consequence, affect antiviral therapy. The use of insulin-sensitizing agents, such as pioglitazone, in HCV treatment increases both SVR and RVR rates [51].

## 6. Diabetes

As with IR, individuals with T2DM are less likely to achieve SVR [52][53]. In addition, diabetics are more likely to experience side effects of the treatment [51]. Compounding this phenomenon, fibrosis progresses more rapidly in diabetics; hence, cirrhosis-free survival rates are significantly lower in diabetics than in nondiabetics [54][55].

## 7. Lipid Profiles

Lipid profiles and virological response to antiviral treatment were recently correlated in many studies. In a recent study from Japan on the lipid fractions (20 subfractions based on particle size) from 44 CHC patients demonstrated that high triglyceride (TG) content of very low-density lipoproteins (VLDL) was strongly predictive of a better response to treatment [56]. In another large European cohort of 575 HCV genotype 1-infected patients, baseline low-cholesterol levels were an independent predictor of a lack of SVR (P = 0.084), but the authors failed to observe any difference in baseline TG levels according to SVR. In spite of the drop in cholesterol level in patients while they were receiving antiviral treatment, cholesterol levels rebounded to either above baseline or to baseline in patients with SVR and nonresponders/relapsers, respectively; (P = 0.02). Triglyceride levels in patients with and without SVR differed only at follow-up (P = 0.028) [57]. A large German, observational, real-world study enrolled 10,390 patients who were treated with PegIFN-α2a/RBV and evaluated and prospectively followed them for cardiovascular and metabolic risk markers, concluding that in contrast to the presence of T2DM, which was a negative predictor of SVR, the presence of elevated total cholesterol levels was associated with higher SVR rates [58].

In another trial that assessed the influence of race on the effect of lipid profiles on SVR, a recent analysis from the Virahep-C study, a prospective study of treatment-naive patients with genotype 1 HCV infection who received PegIFN-α2a/RBV therapy for 48 weeks, observed that pretreatment TG and low-density lipoprotein cholesterol (LDL-C) levels were inversely and directly related to SVR rate, respectively. Yet, baseline LDL-C was significantly higher in Caucasian-Americans compared with African-Americans, although the difference in these parameters did not explain the difference in treatment response between races [59].

There have been 2 recent novel findings from a retrospective analysis of 1604 patients in the IDEAL trial (Individualized Dosing Efficacy vs. Flat Dosing to Assess Optimal Pegylated Interferon Therapy): 1. despite high LDL-C and low HDL-C levels, statin use was independently associated with SVR [60]; and 2. in a genetic analysis of the association between serum lipid levels and hepatic steatosis in CHC patients, Clark and coworkers identified 4 ingle nucleotide polymorphism (SNPs) in the IL28B gene region that were significantly associated with pretreatment LDL (top SNP rs12980275, P = 10-17, good IFN response variant = higher LDL). In a logistic regression model, baseline LDL was strongly associated with SVR. However; including rs12980275 as a covariate attenuated the effect of LDL. Interestingly, this genetic association persisted after treatment for nonresponders, not for patients with SVR. The authors concluded that the poor response with the IL28B variant was strongly associated with lower pretreatment LDL levels and greater hepatic steatosis [61]. In accordance with these findings, another recent trial from Japan showed that the TT genotype of the rs8099917 polymorphism near the IL28B gene was associated with high LDL cholesterol levels, in addition to a high frequency of mutations in the interferon sensitivity-determining region and a wild type of core aa 70; this pattern was the most significant predictive factor of a lack of response to PEG-IFN-α/RBV in genotype 1b CHC patients with high viral loads [62]. These intriguing data may help to provide an explanation for the series of clinical observations linking higher LDL, less steatosis, and SVR.

## 8. Hepatic Steatosis

Hepatic steatosis is commonly observed [63] and is an independent risk factor for disease progression in patients with HCV infection [31]. Various studies have shown that both host and viral factors may contribute to the development of steatosis, with the relative importance of each varying with HCV genotype. In particular, in patients who are infected with genotype 3, steatosis is mostly virus-induced and often severe. In contrast, in those with non-genotype 3, steatosis is mainly associated with host metabolic factors and correlates with body mass index (BMI) and central adiposity [64][65]. It has been shown in large clinical trials that steatosis impairs the response to antiviral therapy [65][66]. The effect, however, is more pronounced in patients with the non-3a genotype [55][56], implicating IR as the mechanism that affects the response to IFN-α and suggesting that viral steatosis does not impair the response to treatment [67][68]. It is worth noting that patients with CHC with virally induced steatosis do not have increased IR levels relative to patients without steatosis [69].

##  9. Menopause and Response to Antiviral Treatment

In recent data, a large cohort of 1000 consecutive patients with compensated liver disease from CHC who were receiving standard PEG-IFN-α/RBV treatment were analyzed for predictors of SVR—menopause correlated with not only more severe liver disease, with high necroinflammatory features, and a high rate of hepatic steatosis but also a low likelihood of SVR in the entire female cohort and in a subgroup of HCV genotype 1 females, probably due to inflammatory factors, such as IL-6 and TNF-α, which modulate at menopause [70]. Despite being upregulated in female patients, these cytokines underwent a further and significant increase at the time of menopause, both circulating and hepatic levels. Ongoing studies are assessing whether the use of hormonal replacement therapy is associated with improved SVRs.

## 10. Host Factors and Individualization of Therapy

**Figure 2:**
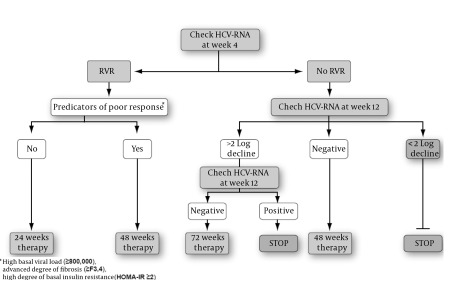
A Proposed Algorithm for Treating Patients With Chronic HCV-4 Bbased on the Kinetics of Viral Response (Response-guided Therapy). RVR, Rapid Viral Response.

In an effort to improve the rates of sustained response to therapy for CHC, various strategies have been used to tailor treatment durations. In addition to depending on treatment responses, recent expert panels have recommended host factors, such as complementary factors, in improving tailoring treatment durations in HCV-4 patients [71] (Figure 2). With the emergence of new data on IL28B, it is expected to be involved in upcoming trials to update these algorithms.In conclusion, besides viral factors, several host factors are responsible for the responsiveness to HCV therapy with Peg-IFN-α/RBV, including host genetic factors, age, ethnicity, gender, menopause, cirrhosis, BMI, steatosis, IR, and T2DM. These factors now are enrolled in all of the algorithms for the individualization of therapy.
